# Ascertaining the Effects of Tissue Sealers on Minor Laparoscopic Procedures between Obstetrics and Gynecology Residents: A Prospective Cohort Study

**DOI:** 10.3390/medicina58050578

**Published:** 2022-04-23

**Authors:** Pasquale De Franciscis, Marco La Verde, Luigi Cobellis, Antonio Mollo, Marco Torella, Fulvio De Simone, Gaetano Maria Munno, Emanuele Amabile, Carla Loreto, Angela Celardo, Nicola Fortunato, Gaetano Riemma

**Affiliations:** 1 Obstetrics and Gynecology Unit, Department of Woman, Child and General and Specialized Surgery, University of Campania “Luigi Vanvitelli”, 80128 Naples, Italy; marco.laverde88@gmail.com (M.L.V.); luigi.cobellis@unicampania.it (L.C.); marcotorella@iol.it (M.T.); fulvdesi@tin.it (F.D.S.); gmm9401@gmail.com (G.M.M.); amabilema93@gmail.com (E.A.); loretocarla@virgilio.it (C.L.); angelacelardo@gmail.com (A.C.); nicola.fortunato@libero.it (N.F.); gaetano.riemma@unicampania.it (G.R.); 2 Gynecology and Obstetrics Unit, Department of Medicine, Surgery and Dentistry “Schola Medica Salernitana”, University of Salerno, 84081 Baronissi, Italy; amollo@unisa.it

**Keywords:** laparoscopy, learning curve, tissue sealers, hemostasis, residents, gynecology

## Abstract

*Background and Objectives*: The type of instrumentation used during laparoscopic surgery might impact on the learning curve of resident surgeons. The aim of this study was to investigate differences in operator satisfaction and surgical outcomes between tissue sealers and classic bipolar instruments during gynecological laparoscopies performed by residents. *Materials and Methods*: A prospective cohort study conducted at two tertiary university hospitals between March 2019 and March 2021, on consecutive procedures: salpingo-oophorectomies (Group 1) and salpingectomies (Group 2), subdivided according to the utilized device: radiofrequency tissue sealers (Groups A1 and A2) or bipolar forceps (Groups B1 and B2). *Results*: 80 procedures were included. Concerning salpingo-oophorectomies, better visibility (8.4 ± 0.8 vs. 7.3 ± 0.9; *p* = 0.03), reduced difficulty (5.4 ± 1.2 vs. 7.0 ± 1.4; *p* = 0.02), improved overall satisfaction (9.2 ± 0.4 vs. 7.6 ± 1.0; *p* = 0.02) and reduced procedure time (7.8 ± 3.4 vs. 12.6 ± 3.1; *p* = 0.01) were reported by residents using tissue sealers. Intraoperative blood loss (12.2 ± 4.7 mL vs. 33.2 ± 9.7 mL; *p* = 0.01) and 24 h postoperative pain (4.5 ± 1.1 vs. 5.7 ± 1.8; *p* = 0.03) were lower in group A1 than B1. For salpingectomies, a significant reduction in duration was found in A2 compared to B2 (7.2 ± 3.4 min vs. 13.8 ± 2.2 min; *p* = 0.02). Tissue sealers enhanced visibility (8.1 ± 1.1 vs. 6.7 ± 1.4; *p* = 0.01), difficulty (6.5 ± 1.1 vs. 7.5 ± 0.9; *p* = 0.04) and improved satisfaction (9.3 ± 0.5 vs. 7.5 ± 0.6; *p* = 0.01). Moreover, hemoglobin loss and postoperative pain were reduced in A2 relative to B2 [(8.1 ± 4.2 % vs. 4.5 ± 1.1%; *p* = 0.02) and (5.1 ± 0.9 vs. 4.1 ± 0.8; *p* = 0.03), respectively] *Conclusions*: The use of sealing devices by residents was related to reduced difficulty as well improved visibility and overall satisfaction, with improved surgical outcomes.

## 1. Introduction

During recent times, gynecological surgery and training has experienced many radical changes, including the reduction in operative cases due to the COVID-19 pandemic [1]. For residents in gynecological surgery, this means fewer cases being assigned to the operating room [2].

Basic surgical ability and knowledge can be practiced in simulation laboratories before operating on the patient [3]. However, current residents’ training is basically focused on anatomy and surgical techniques first, meanwhile, the application in the surgical field of the different instrumentation and techniques for coagulation and hemostasis are still less considered. Such issues would increase patient safety. In fact, coagulation, vessel sealing and control of hemostasis are crucial steps for every surgical procedure [4]. For the improvement of safety and precision, over the last two decades, several devices for laparoscopic use have been developed. Basically, these instruments belong to two major categories: bipolar forceps or tissue sealers [4,5]. Bipolar forceps are considered the basic electrosurgical instruments, not only due to their increased safety profile relative to monopolar electrosurgery, but also due to their feasibility in terms of costs and benefits, since they are reusable and available worldwide [6,7].

On the other hand, tissue and vessel sealers are updated and safe instruments, since they feature the real-time identification of the tissue to be cut. For this reason, sealers allow a uniform compression of tissues and vessels, essential for satisfying surgical outcomes [4,5,8].

With regard to safety, bipolar forceps and tissue sealers were mainly reported comparable, with the possibility of tissue sealers allowing cut and coagulation simultaneously, without the need for switching instruments during the procedure. Tissue/vessel sealers take advantage of the combination of pressure granted by the handpiece, and a source of energy (traditionally radiofrequency or ultrasound), applied to the target tissue for tissue synthesis [4].

Several studies have addressed the impact of these technologies on different kinds of general and specialized surgeries, showing similar data regarding efficacy and safety. However, there is a scant amount of evidence regarding the possible benefits or disadvantages of such categories of devices in gynecologic laparoscopy, although a minimally invasive laparoscopy represents the gold standard approach in over the 70% of procedures for uterine and adnexal benign pathologies [8].

Moreover, the current literature analyzes the advantages and disadvantages of the several hemostatic devices solely for expert surgeons, without considering that the use of tissue sealers or bipolar forceps by resident surgeons might achieve different results. In fact, minor surgeries, including laparoscopic adnexectomy and salpingectomy, are the most common interventions carried out by residents as a first surgeon [3,9].

Based on this, the aim of this study was to evaluate whether the use of a hemostatic surgical device impacts on the learning curve and surgical outcomes of gynecology residents performing minor laparoscopic procedures.

## 2. Materials and Methods

This study was designed as a prospective cohort study conducted at two centers related to the University of Campania “Luigi Vanvitelli” (the Obstetrics and Gynecology Unit of the AOU L. Vanvitelli, Naples, Italy, and the Obstetrics and Gynecology Unit, AORN Sant’Anna e San Sebastiano, Caserta, Italy) between March 2019 and March 2021.

The Institutional Review Board (IRB) of the University of Campania “Luigi Vanvitelli” approved the study with protocol no. 712/15-11-19, with date 15 November 2019. All patients signed a written informed consent form describing the surgical approach, common complications, as well as privacy and anonymity protection throughout data collection and analysis.

Senior gynecology residents who served as first surgeons during the entire laparoscopic salpingo-oophorectomy or salpingectomy were considered. In order to maintain consistency, junior residents, fellows and consultants were excluded from the analysis.

Patients referred from the outpatient gynecological clinics of our unit who, according to national and international treatment guidelines, were eligible for a planned laparoscopic salpingectomy or salpingo-oophorectomy for benign gynecological pathologies were enrolled. The definition of a benign gynecological pathology included an ultrasonographic suspicion of a unilateral tubal, ovarian, or tubo-ovarian pathology, sized between 3 to 7 cm in its largest diameter, without local extension. Confirmation of the benignity of the diseases was achieved through postsurgical histopathological examination.

Women who were not suitable for or denied a laparoscopic approach, declined the procedure, did not sign a written informed consent form, were suffering from a gynecologic malignant disease or severe systemic illnesses (i.e., autoimmune or endocrine diseases, severe coagulopathy or cardiac pathology) and for whom procedures were converted to laparotomy, were excluded from the analysis.

Consecutively enrolled patients were divided in two main categories depending on which device was chosen for the entire procedure:

- Group A: a 5 mm diameter, 35 cm length curved branch radiofrequency tissue sealer connected to a dedicated electric generator (Enseal NSGL2, Ethicon Endo-Surgery, Germany or Ligasure, Medtronic, USA).
- Group B: 5 mm diameter, 35 cm classic rotating bipolar forceps (RoBi Kelly, Karl Storz Endoskope, Germany).

Therefore, patients from each category were subdivided in accordance with the type of surgical procedure: groups A1 and B1 for laparoscopic salpingo-oophorectomy, and groups A2 and B2 for laparoscopic salpingectomy.

Operative procedures were carried out according to the most recent national guidelines and following the good clinical practice of our operative unit. No changes from a routine salpingectomy or salpingo-oophorectomy were actuated. The same set of senior residents acted as operators for both groups A and B.

### 2.1. Primary and Secondary Outcomes

The primary outcomes of interest were regarding the subjective evaluation of the surgical procedure by the resident serving as a first operator, by means of a Numeric Rating Scale (NRS):

(i). Vision of the surgical field (considering 0 for “inadequate vision” and 10 for “optimal vision”)
(ii). Interpretation of the difficulty of the intervention (considering 0 for “extremely easy” and 10 for “extremely difficult”)
(iii). Overall procedural satisfaction (considering 0 for “complicated or incomplete procedure” and 10 for “uncomplicated and satisfying intervention”)

Several secondary outcomes concerning intraoperative and postoperative characteristics were investigated:

(i). Procedure time, defined as vascular time interval, expressed in minutes, regarding devascularization and removal of the targeted organ. For laparoscopic salpingectomy, the recorded interval included the resection of the isthmic part of the tube and the tubal arterial arch. Regarding salpingo-oophorectomy, in addition to the abovementioned interval, the recorded time included the resection of the infundibulopelvic and the round ligaments.
(ii). Postoperative hospitalization days, defined as the number of days between procedure and discharge
(iii). Hemoglobin (Hb) percentage variation expressed as the rate between presurgical and first postsurgical Hb.
(iv). Intraoperative and postoperative complications (i.e., blood transfusion necessity, laparotomic conversion, infection or other common complications)
(v). 24 h postoperative pain, expressed using a 0–10 NRS, in which 0 was intended as “no pain” and 10 was “extremely painful”). Patients from both groups underwent the same postoperative analgesic treatment according to our institution’s protocol.

### 2.2. Sample Size

According to the current literature, assuming an A-priori calculation for the size of sample required to detect a significant difference between the groups, given 80% power and an alpha level of 0.05, was 40 procedures per arm of the study, including a 7% anticipated opt-out rate.

### 2.3. Statistical Analysis

Statistical analysis was conducted using Stata 14.1 (StataCorp, College Station, TX, USA). Normally distribution of data was measured using the Shapiro–Wilk test. Continuous variables were reported as means and standard deviations (SDs). Dichotomous data were reported as the absolute number and percentages. For continuous variables, differences were evaluated by means of the t-test, while differences in the proportions between the groups were analyzed using the Fisher’s exact and Chi-squared test, where appropriate. Association between the instrument used and the outcomes of interest was assessed using a risk ratio (RR) with 95 % confidence intervals (CIs). Statistical significance was set as a *p* < 0.05.

## 3. Results

A total of 80 minor surgical procedures matched the inclusion criteria and were evaluated. Of those, 40 were laparoscopic salpingo-oophorectomies: 21 were carried out with a tissue sealer (group A1) while 19 were carried out with bipolar forceps (B1). Forty salpingectomies were undertaken: 20 with a tissue sealer (A2) and 20 with bipolar forceps (B2) (Table 1).

There were no differences concerning the largest diameter of the lesion between groups A and B (4.8 ± 2.1 cm vs. 5.4 ± 1.7 cm; *p* = 0.66), and there was no mass extending outside the ovary or the tube.

### 3.1. Visualization and Satisfaction

Concerning the salpingo-oophorectomies, residents’ judgment underlined an enhanced visibility of the surgical field with the use of a tissue sealer rather than bipolar forceps (8.4 ± 0.8 vs. 7.3 ± 0.9; *p* = 0.03). The intervention was judged to be easier with a radiofrequency device than with bipolar forceps (5.4 ± 1.2 vs. 7.0 ± 1.4; *p* = 0.02). Moreover, an improved overall satisfaction was reported by residents in the tissue sealer group, than in the bipolar forceps group (9.2 ± 0.4 vs. 7.6 ± 1.0; *p* = 0.02). Consequently, the mean procedure time was reduced with the use of a tissue sealer (7.8 ± 3.4 vs. 12.6 ± 3.1; *p* = 0.01) (Table 2). Compared to standard bipolar forceps, the use of a tissue sealer in salpingo-oophorectomies showed a reduced RR for procedure time over 10 min (RR 0.36; 95% CI 0.17 to 0.73; *p* = 0.01), a reduced RR for visibility of the surgical field lower than six points (RR 0.45; 95% CI 0.18 to 0.87; *p* = 0.03), a reduced risk of a difficulty score over six points (RR 0.40; 95% CI 0.21 to 0.67; *p* = 0.02) and a reduced RR for overall satisfaction under six points (RR 0.33; 05% CI 0.19 to 0.74; *p* = 0.02).

With regard to the salpingectomies, a statistically significant reduction in the procedure duration was notable while using a tissue sealer rather than bipolar forceps (7.2 ± 3.4 min vs. 13.8 ± 2.2 min; *p* = 0.02). When a tissue sealer was used, enhanced visibility of the surgical field was achieved (8.1 ± 1.1 vs. 6.7 ± 1.4; *p* = 0.01), together with a reduction in procedural difficulty (6.5 ± 1.1 vs. 7.5 ± 0.9; *p* = 0.04) and a better overall operator satisfaction (9.3 ± 0.5 vs. 7.5 ± 0.6; *p* = 0.01) was observed (Table 3). Relative to standard bipolar forceps, the use of a tissue sealer in laparoscopic salpingectomies showed a reduced RR for an overall procedure time longer than 10 min (RR 0.56; 95% CI 0.22 to 0.81; *p* = 0.02), a reduced RR for vision of the surgical field with a score under six points (RR 0.21; 95% CI 0.14 to 0.49; *p* = 0.01), reduced risk of a difficulty score over six points (RR 0.84; 95% CI 0.76 to 0.91; *p* = 0.04) and a reduced RR for an overall satisfaction score of less than six points (RR 0.30; 05% CI 0.15 to 0.55; *p* = 0.01).

### 3.2. Surgical Outcomes

Comparing tissue sealer-based to bipolar-forceps-based salpingo-oophorectomy, a significant reduction in intraoperative blood loss was found (12.2± 4.7 mL vs. 33.2 ± 9.7 mL; *p* = 0.01). Postoperative pain was lower with tissue sealer usage (4.5 ± 1.1 vs. 5.7 ± 1.8; *p* = 0.03). No other differences were observed between the intraoperative data. For each group, there were no surgical complications or re-interventions (Table 2). Compared with standard bipolar-based salpingo-oophorectomy, using a tissue sealer was related to a reduced risk of intraoperative blood loss over 20 mL (RR 0.54; 95% CI 0.21 to 0.76; *p* = 0.01) and a reduced risk of postoperative pain over six points after 24 h (RR 0.67; 95% CI 0.51 to 0.89; *p* = 0.02) There were no differences regarding the percentage of Hb loss after 24 h over 7% (RR 0.90; 95% CI 0.71 to 1.15; *p* = 0.24) and the number of post-surgical complications (RR 1.15; 95% CI 0.15 to 4.55; *p* = 0.89).

The evaluation of laparoscopic salpingectomies reported that the use of a tissue sealer had a reduced percentage of Hb loss 24 h after the intervention (8.1 ± 4.2 vs. 4.5 ± 1.1%; *p* = 0.02). Moreover, postoperative pain at 24 h was reported to be higher in procedures carried out with bipolar forceps (5.1 ± 0.9 vs. 4.1 ± 0.8; *p* = 0.03). The other investigated outcomes were comparable between the two groups (Table 3). Compared to a standard approach using bipolar forceps, tissue-sealer based salpingo-oophorectomies performed by residents showed a reduced risk of the percentage of Hb loss after 24 h being over 7% (RR 0.78; 95% CI 0.65 to 0.90; *p* = 0.02), and a reduced RR for postoperative pain over six points after 24 h (RR 0.69; 95% CI 0.58 to 0.84; *p* = 0.02). No differences were reported concerning intraoperative blood loss over 20 mL (RR 0.54; 95% CI 0.21 to 0.76; *p* = 0.01) and intraoperative complications (RR 0.92; 95% CI 0.21 to 3.77; *p* = 0.94).

## 4. Discussion

This study on consecutive minor gynecologic laparoscopic procedures carried out by senior residents suggests that the use of a radiofrequency tissue sealer, instead of bipolar forceps, was associated with increased satisfaction, better visibility and a reduced procedure time. Moreover, intraoperative and postoperative blood losses were significantly reduced with the use of a tissue sealer.

Minimally invasive surgery involves less surgical trauma, faster hospital discharge and decreased postoperative pain, with less time needed for returning to a normal life routine [10,11,12,13,14]. It is essential to teach and train laparoscopy in every surgical residency, especially in gynecological surgery, in which a less invasive approach significantly improves the postsurgical quality of life of both fertile-age and postmenopausal women [13,15,16,17,18].

Several lines of evidence showed that a standardized, step-by-step, learning program approach is mandatory to achieve basic surgical skills [19,20].

The early phase of the laparoscopy learning curve can be achieved in a simulation laboratory, allowing for teaching and practice in a safe, systematic and unharmful environment [21].

However, in the second path of the learning curve, the training field is switched into the operating room. In such a scenario, choosing the right instrumentation is a crucial step to improve the surgical skills of young surgeons [21,22,23,24]. When a tissue sealer was used, the residents not only predictably reported improved surgical outcomes and an enhanced procedure time, but overall satisfaction improved and surgical field visibility was also easier during the procedure itself. It should be noted that a comfortable environment enhances both the learning path and the intraoperative and postoperative outcomes.

This study shows several strengths. First, it gives insights about the perception of surgical field vision and the satisfaction of gynecology residents, which is important in the assessment of a global, standardized, surgical skill program that needs to also involve the resident surgeons in the didactic path, in order to maximize the learning program and simplify the laparoscopic learning curve. Moreover, the prospective analysis of the surgical procedures represents the best available option to reduce selection biases, since, due to ethical constraints, it was not possible to randomize the instrumentation (tissue sealer or bipolar forceps) to be used during the procedures. Conversely, this study has several limitations. The first is related to the small number of procedures available for analysis. For this reason, a sub-analysis concerning residents age, sex and skill status of the investigated outcomes was not performed. In addition, in our institution, salpingo-oophorectomies and salpingectomies are exclusively performed by gynecologists, avoiding the possibility of performing comparisons between gynecological and general surgical or other surgical residencies.

## 5. Conclusions

The use of tissue sealers rather than standard bipolar forceps during laparoscopic salpingectomies or salpingo-oophorectomies, by gynecology residents as first surgeons, was related to reduced difficulty, as well as improved visibility and overall satisfaction. Moreover, there was there was significantly less post-surgical pain and blood loss in the tissue sealer group than in the bipolar forceps group. Choosing the adequate instrumentation during the surgical learning path should be considered a critical step for the surgical training.

## Figures and Tables

**Table 1:** Baseline characteristics of women undergoing laparoscopic interventions by residents.

	A1 Adnexectomies with Tissue Sealer	B1 Adnexectomies with Bipolar Instruments	A2 Salpingectomies with Tissue Sealers	B2 Salpingectomies with Bipolar Instruments
Age (mean ± standard deviation)	54.8 ± 5.9	52.9 ± 4.5	33.3 ± 7.0	35.3 ± 4.3
History of laparotomies	0/21 (0%)	0/19 (0%)	0/16 (0%)	0/14 (0%)
BMI (Kg/m2)	22.1 ± 6.0	21.5 ± 5.2	21.8 ± 3.1	22.9± 5.6

**Table 2:** Primary and secondary outcomes for laparoscopic salpingo-oophorectomies.

	Tissue Sealer (A1) *n* = 21	Classic Bipolar (B1) *n* = 19	*p*-Value
Procedure Time (mins)	7.8 ± 3.4	12.6 ± 3.1	0.01 *
Vision of Surgical Field (NRS)	8.4 ± 0.8	7.3 ± 0.9	0.03 *
Difficulty Score (NRS)	5.4 ± 1.2	7.0 ± 1.4	0.02 *
Overall Satisfaction (NRS)	9.2 ± 0.4	7.6 ± 1.0	0.02 *
Intraoperative Complications	0/21	0/19	0.99
Intraoperative blood-loss (mL)	12.2 ± 4.7	33.2 ± 9.7	0.01 *
24 h Hb-Loss (%)	8.9 ± 1.0	8.2 ± 3.2	0.09
Post-Surgical Complications	1/21	0/21	0.97
24 h Postoperative pain (NRS)	4.5 ± 1.1	5.7 ± 1.8	0.03 *

* *p*-value < 0.05.

**Table 3:** Primary and secondary outcomes for laparoscopic salpingectomies.

	Tissue Sealer (A2) *n* = 20	Classic Bipolar (B2) *n* = 20	*p*-Value
**Procedure Time (mins)**	7.2 ± 3.4	13.8 ± 2.2	0.02 *
**Vision of Surgical Field (NRS)**	8.1 ± 1.1	6.7 ± 1.4	0.01 *
**Difficulty Score (NRS)**	6.5 ± 1.1	7.5 ± 0.9	0.04 *
**Overall Satisfaction (NRS)**	9.3 ± 0.5	7.5 ± 0.6	0.01 *
**Intraoperative Complications**	0/16	1/14	0.81
**Intraoperative blood-loss (mL)**	17.1 ± 5.6	16.8 ± 3.2	0.74
**24 h Hb-Loss (%)**	4.5 ± 1.1	8.1 ± 4.2	0.02 *
**Post-Surgical Complications**	0/16	0/14	0.99
**24 h Postoperative pain (NRS)**	4.1 ± 0.8	5.1 ± 0.9	0.02 *

* *p*-value < 0.05.

## Data Availability

The data presented in this study are available on request from the corresponding author.
